# Care for Superannuated Physicians

**Published:** 1916-07

**Authors:** 


**A Monthly Review of Medicine and Surgery**

EDITOR AND PUBLISHER, DR. A. L. BENEDICT, 228 Summer St., corner of Elmwood Ave., Buffalo, (Address for all communications. Please make personal and telephone calls before 1 P. M.)

**Third, in age, on the western continent. Independent, but supports professional organization. News limited mainly to Western N. Y. and Penn.**

**Subscription $2.00 a year; $3.00 for two years in advance. Changes and errors in address or failure or duplication of delivery, should be reported immediately.**

Yearly Volume 71 JULY, 1916. Number 11

### Care for Superannuated Physicians.

We feel keenly that definite provision should be made for physicians and their families, who are in need on account of old age, sickness or any other cause. The analogy between the clergy and the medical profession is commonly emphasized in speeches on appropriate occasions. Are not the two professions in reality close enough in their service to humanity, so that the analogy may be extended to such provision against want? For example, one of the protestant denominations is raising an endowment fund of several million dollars, representing an average per capita tax on its members of almost exactly $7, about $2 more than the average per capita tax of the state of New York. The fund will represent just about $1000 per minister and it is obvious that the interest on this amount, dispensed as needed, or even paid to an insurance company for an old-age and disability annuity, is adequate to provide against actual suffering. When we consider further that these figures imply one minister to about 150 church members, it is obvious that the fund is far higher than would have been necessary if any reasonable economic principle had been applied to the ordination of clergymen.

At the outset, a difficult problem is encountered. The clergy have a well organized, definite association of laymen, trained to give for church purposes in general. On the contrary, the bond between the physician and his clientele is much less firm, even when the physician considers himself a "family doctor." The influence of the physician in securing contributions for medical philanthropy from which he derives no direct benefit and in regard to which he may be absolutely disinterested, is much smaller, measured in dollars, than the corresponding influence of the clergy. So far as we are aware, no appeal has ever been made to the laity, for benevolence to physicians themselves, considered as members of a profession that is largely altruistic. Of course, physicians as individuals, have received such benevolence on account of family, personal, fraternal and other affiliations or simply as paupers. To what extent will the laity contribute to the support of needy physicians simply on the ground of their having exercised a function in regard to society in general, that compares closely with that of the clergy? This question suggests another. Without in any way implying that the clergy should have taken the same course, it is conceivable that the medical profession may feel that they themselves will assume the burden of providing for their own professional colleagues or at least that they will accept only such contributions from laymen as are absolutely voluntary and come from such large fortunes or bequests as to represent no sacrifice of self or dependents of the donor.

Such questions cannot be answered entirely from the standpoint of pride; the actual ability to meet demands must be considered. Quite accurate statistics of clerical incomes are available. The minimum ministerial salary allowed at present in many subdivisions of various religious denominations is $1000. Past estimates show salaries down to half of this amount but it must be conceded that the low estimates apply mainly to clergymen who have not in any sense complied with the formal educational requirements and training of their own profession or any other, by analogy. Men of the same grade of preparation for healing bodily ills would scarcely be included in the medical profession at present; although an occasional obituary with "years of practice" instead of the name of a medical school and date of graduation, shows that the analogy has held good in the past. Frequently quoted averages of net medical incomes agree closely with the figures set for the clergy but we are inclined to believe that such statistics are practically incorrect, perhaps because of the inclusion of incomes at or near the zero point during the inevitable stage of post graduate medical study, in hospitals, etc., and during the equally inevitable though regretable period of establishing a practice. Considered a priori, it would seem that moderate senile or other physical disability should interfere quite as much with the ability to earn a living by medical practice as by performing ministerial duties, yet observation seems to show that there are more clergymen unable to hold positions and totally dependent, than there are physicians.

In one respect, the problem is simpler than for the clergy. The multiplicity of religious denominations in this country, even excluding those of small numbers and representing immigration of decidedly foreign peoples, whom we would term "heathen" with no implied discourtesy, is almost inconceivably large. Even counting such denominations of Christians as are numerous, running beyond the million mark, at least a dozen organizations of benevolence, are practically necessary. On the contrary, the medical profession, with a reasonable insistance on conformity to accepted ethical customs, is already practically unified, at least for any such problem as we are considering.

The first practical step toward adequate provision for needy members of the medical profession is to secure some basis for estimates of what would be necessary. We therefore ask our readers to reply to the following questions, anonymously, if preferred:

1. Do you favor or oppose systematic provision for superannuated and disabled physicians?

2. If favorable, should such provision be made solely by the medical profession or should lay assistance be invoked, and to what extent?

3. Do you yourself need such assistance at present and, if so, on account of old age or what other cause?

4. So far as you can judge, what are your own chances of requiring such assistance, in case of surviving past the period of work, or for disability otherwise arising, with due regard to insurance, probable support by children and other means of support?

5. Would you contribute for such a fund, to what amount, do you advise raising a fund annually as required or the establishment of an endowment?

6. Aside from demonstration of need of such a degree as to warrant relief, would you advocate any special requirement beyond the possession of a medical degree and bona fide practice along lines generally recognized as ethical?

7. Please make any additional suggestions.

We may say that we have no interested motives in agitating this matter and that we are willing to co-operate to any reasonable extent in caring for unfortunate and deserving members of the profession.

